# Prior Signal Acquisition Software Versions for Orbitrap Underestimate Low Isobaric Mass Tag Intensities, Without Detriment to Differential Abundance Experiments

**DOI:** 10.1021/acsmeasuresciau.1c00053

**Published:** 2022-03-09

**Authors:** Tom S. Smith, Anna Andrejeva, Josie Christopher, Oliver M. Crook, Mohamed Elzek, Kathryn S. Lilley

**Affiliations:** † MRC Toxicology Unit, University of Cambridge, Cambridge CB2 1QR, U.K.; ‡ Department of Biochemistry, University of Cambridge, Cambridge CB2 1QW, U.K.; § Department of Statistics, University of Oxford, Oxford OX1 3LB, U.K.

**Keywords:** Orbitrap, notch, tandem mass tagging, quantitative proteomics, reporter ion quantification, benchmark

## Abstract

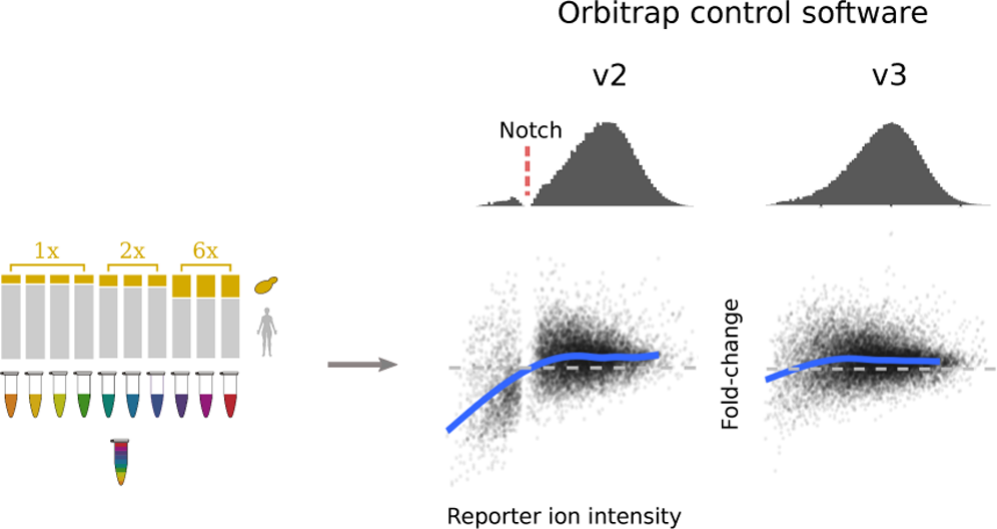

Tandem mass tags (TMTs) enable simple and accurate quantitative proteomics for multiplexed samples by relative quantification of tag reporter ions. Orbitrap quantification of reporter ions has been associated with a characteristic notch region in intensity distribution, within which few reporter intensities are recorded. This has been resolved in version 3 of the instrument acquisition software Tune. However, 47% of Orbitrap Fusion, Lumos, or Eclipse submissions to PRIDE were generated using prior software versions. To quantify the impact of the notch on existing quantitative proteomics data, we generated a mixed species benchmark and acquired quantitative data using Tune versions 2 and 3. Intensities below the notch are predominantly underestimated with Tune version 2, leading to overestimation of the true differences in intensities between samples. However, when summarizing reporter ion intensities to higher-level features, such as peptides and proteins, few features are significantly affected. Targeted removal of spectra with reporter ion intensities below the notch is not beneficial for differential peptide or protein testing. Overall, we find that the systematic quantification bias associated with the notch is not detrimental for a typical proteomics experiment.

## Introduction

Bottom–up quantitative proteomics entails proteolytic digestion of proteins to peptides, quantification of peptide abundances by mass spectrometry (MS), and summarization of protein abundances. Data-dependent acquisition (DDA) is the classical acquisition mode. Due to the stochastic nature of peptide selection, not all peptides present in a sample are fragmented and sequenced, with data completeness diminishing as the number of samples increases.^1^ Missing values are reduced with data-independent acquisition (DIA) approaches,^2^ although there are still detection threshold limits and samples cannot be multiplexed in typical DIA workflows.

Alternatively, samples may be labeled with isobaric tags that possess the same mass, but different distributions of heavy isotopes within the tags, such that a sample-specific mass reporter tag is released by high-energy collision-induced dissociation.^3^ By enabling sample multiplexing, the frequency of missing values is thus reduced compared to label-free quantification.^4^ Furthermore, all samples are quantified from the same peptide spectrum matched (PSM) ions, greatly simplifying summarization to protein-level abundances.^5^

Tandem mass tags (TMTs) are the most commonly used isobaric tags, with current chemistry allowing up to 18 samples to be multiplexed.^6^ Since quantification is typically of the tag rather than the peptide, TMT proteomics has been shown to suffer ratio compression by the presence of co-isolated “interference” peptides.^7^ Such compression can be partially mitigated by the use of synchronous precursor selection (SPS)-MS3 quantification.^8^ Thus, robust quantification of TMT-labeled peptides requires high-resolution, accurate mass spectrometers, with Orbitrap devices being commonly employed. Intriguingly, a recent characterization of TMT reporter ion signals obtained from Orbitrap identified a consistent lack of detected reporter ion intensities within a specific range, which visually appears as “notch” in the distribution of intensities.^9^ The presence of the notch was determined to depend on the automatic gain control (AGC) target and maximum injection time Orbitrap parameters, with higher values reducing its prominence. Hughes et al. hypothesized that the cause of the notch is rooted in the signal processing behavior, from their observation of a notch in all datasets acquired via Orbitrap-based measurements, regardless of the other associated MS hardware, and the exhaustive consideration of user-defined parameters. Hughes et al. further speculated that standard procedures to remove low-intensity spectra would mitigate any potential issues with quantification inaccuracies.

The release notes for the Orbitrap Fusion Series 3.0 signal acquisition software, Tune, explain that the notch has been resolved in Item DE 54684: “Addressed the peak intensity (linearity) for extremely low S/N values”.^10^ Nevertheless, published datasets have used prior software versions and trust in the results from these experiments is contingent upon accurate quantification.

Here, we further examine the “notch” phenomenon and demonstrate what happens to reporter ion intensities that fall inside the notch. Crucially, we examine the overall impact of the notch for the detection of significant fold changes and estimation of their magnitude in an experiment that aims to measure differential abundance.

## Results and Discussion

**Figure 1:**
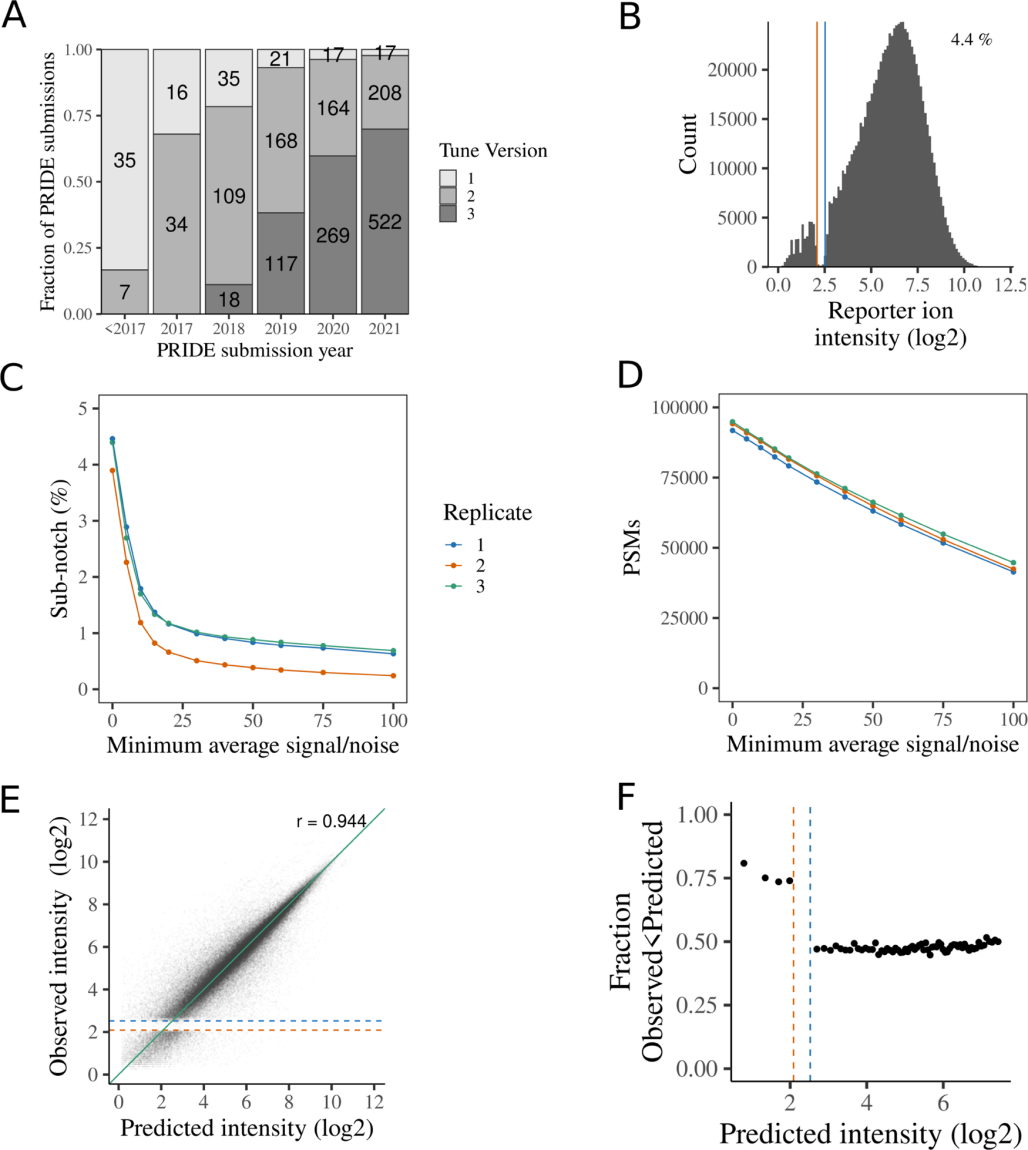
(A) Tune major versions for Orbitrap Lumos, Fusion, and Eclipse PRIDE submissions. (B) Distribution of ion signals for TMT reporters U-2 OS LOPIT-DC, replicate 1. The percentage of tag intensities below the upper boundary of the notch is stated in the top right corner. (C, D) Impact of filtering PSMs by their average signal/noise. (C) Proportion of reporter tag intensities at or below the notch. (D) Number of PSMs remaining. (E) Observed tag intensities vs predicted tag intensities (log2 scaled). The green line is equality. The Pearson product-moment correlation coefficient is shown in the top right corner. (F) Fraction of observed tag intensities that are below the prediction for binned predicted tag intensity. Observed tag intensities below the notch are predominantly underestimated relative to the prediction. The approximate boundaries of the notch region are denoted by vertical or horizontal lines in (B), (E), and (F). Equivalent plots for replicates 2 and 3 are shown in Figures S1 and S2.

To estimate the proportion of published Orbitrap datasets using Tune versions that will generate a notch, we downloaded.raw files from PRIDE for all studies using only Orbitrap Fusion, Lumos, or Eclipse hardware since these use the same series of acquisition software. We then extracted the version of Tune used from the.raw file (see methods). In total, 1283 studies were examined, with the remaining studies either falsely stating the instrument model or not possessing files that could be parsed for meta data (Figure 1A). Overall, 47.3% of studies used Tune versions that generate a notch, including 30.1% of submissions in 2021. It is therefore imperative to determine what impact the notch has on quantitative proteomics.

We first reanalyzed previously published Orbitrap SPS-MS3 TMT data acquired using Tune v2 to demonstrate that average signal/noise filtering is not a sufficient remedy for the notch. Between 3.9 and 4.5% tag intensities fell within or below the notch (Figures 1B and S1b,c) in our U-2 OS LOPIT-DC^11^ experiments, with the proportion varying considerably between tags within a given experiment (Figure S1d–f). Using increasingly stringent filtering to remove PSMs with low average signal/noise ratios reduces the proportion of values below the notch (Figure 1C,D). However, even after removing PSMs with average signal/noise less than 100, 0.24–0.69% of remaining tag intensities are below the notch, while 52.8–55.0% of quantified PSMs are discarded. Using a more moderate 10-fold filter, as previously suggested,^12^ removes 6.6–6.8% of the quantified PSMs, with 1.2–1.8% of the remaining reporter intensities being below the notch. Thus, while average signal/noise filtering will increase quantification accuracy, it does not completely remove intensities below the notch.

To demonstrate what happens to notch intensities, we used redundant PSMs to predict expected reporter ion intensities. In brief, we considered sets of PSMs from the same peptide sequence and used the reporter ion intensities from the highest-intensity PSM to predict the expected reporter ion intensity values for the other PSMs (see Experimental Section). Predicted and observed tag intensities were highly correlated, confirming the validity of this approach (Figures 1E and S2a,b). As expected, intensities below the notch are predominantly underestimated relative to the prediction (Figures 1F and S2c,d). Thus, we demonstrate that the issue addressed with Tune 3.0 regarding “peak intensity (linearity) for extremely low S/N values” manifests as discontinuinty (“notch”) in the reporter ion intensity distribution. Values that should fall in the notch are not missing, they are systematically underestimated.

**Figure 2:**
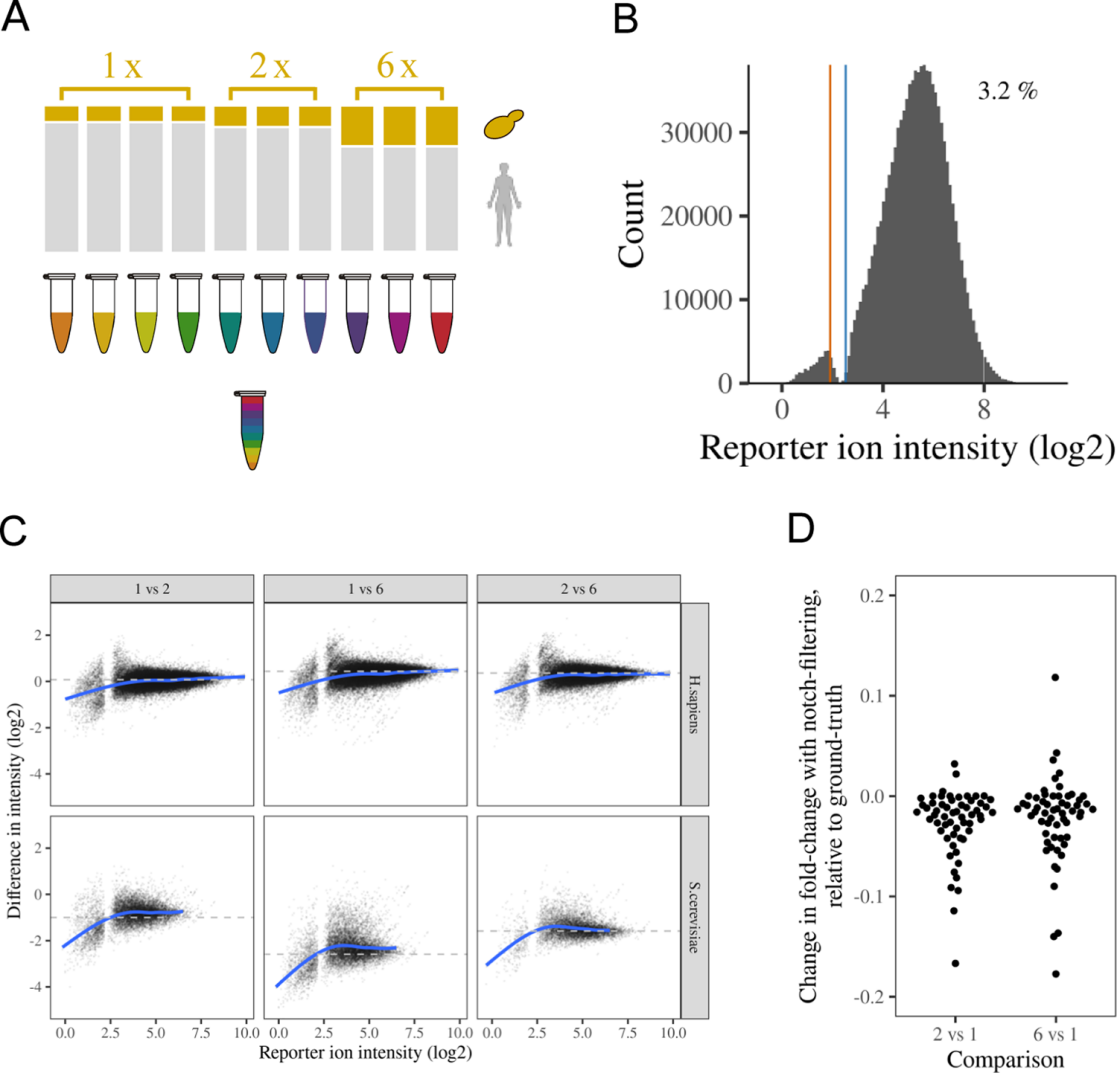
Impact of the notch on a differential expression benchmark proteomics experiment. (A) Schematic representation of the benchmark TMT proteomics experimental design. Yeast peptides were spiked into human peptides at 1×, 2×, and 6× volumes to induce ground truth fold changes for both yeast and human proteins and labeled with TMT. (B) Distribution of reporter ion intensities for TMT reporters. The approximate boundaries of the notch region are denoted by vertical lines. The percentage of tag intensities below the upper boundary of the notch is stated in the top right corner. (C) Difference between a single tag intensity and the mean tag intensity for a comparator group of tags. The ground truth is denoted by a dashed horizontal line. The blue line presents a generalized additive smoothing model for the relationship between tag intensity and intensity difference. (D) Difference in fold change when including notch filtering, relative to the ground truth. Positive values represent fold-change estimates closer to ground truth upon notch filtering. Only yeast proteins with at least one reporter ion intensity below the notch are shown.

To explore the impact of the notch on a typical quantitative TMT MS3 proteomics experiment, we considered suitable published benchmark experiments. Hughes et al. performed a spike-in benchmark experiment to assess the impact of the notch.^9^ However, this included only 550 spike-in peptides, precluding a consideration of the impact of the notch in a routine proteomics experiment. In another benchmarking study, O’Connell et al. spiked peptides from *Saccharomyces cerevisiae* cell lysates into *Homo sapiens* peptide samples at known concentrations, thus mimicking a control vs treatment(s) differential protein abundance experimental design.^4^ However, the authors used a relatively high AGC target of 150 000 and a maximum injection time of 150 ms. Thus, only 0.6% tag intensities were observed at or below the notch, preventing any analysis of the impact of the notch in a more typical setting (Figure S3a). We therefore created our own spike-in benchmark experiment, aiming to observe a notch with typical prominence (see the Methods section). Proteins extracted from whole cell lysates of *S. cerevisiae* and *H. sapiens* osteosarcoma cell line U-2 OS were digested to peptides and mixed, such that *S. cerevisiae* peptides were at 1× (5 μg), 2× (10 μg), and 6× (30 μg) volumes with *H. sapiens* peptides making each sample up to 100 μg of the peptide (Figure 2A). Thus, we generated differences in protein abundance between 1.06- and 6-fold for *H. sapiens* and *S. cerevisiae* proteins. Peptides were then labeled with TMT and quantified on an Orbitrap Lumos Mass Spectrometer with Tune 2.1 and Tune 3.4, with an AGC target of 50 000 and a max injection time of 120 ms, which we expected to yield a moderate and typical notch^9^ (see the Methods section for details). Using these parameters and Tune 2.1, 3.2% of tag intensities were within or below the notch (Figure 2B), with *S. cerevisiae* peptides showing a slightly greater proportion of low tag intensities (Figure S3b,c). In total, we obtained 109 779 PSMs from 10 982 protein groups, of which 8335 and 2469 could be assigned to *H. sapiens* and *S. cerevisiae*, respectively.

We applied strict filtering to minimize the possibility of PSM mis-identification or interference, demanding co-isolation <10%, and Sequest Delta CN score >0.5, with the latter ensuring that rank 2 peptide matches for a given spectrum have a score less than half the rank 1 peptide match (see the Methods section). We further removed PSMs with average reporter signal/noise <10. Using these thresholds, 19 975 PSMs were retained. As expected, tag intensities below the notch were clearly underestimated, with the correct fold change only observed when reporter ion intensities were above the notch (Figure 2C). In contrast, using Tune 3.4, no notch was visible (Figure S4a) and the underestimation of fold changes at low reporter ion intensity was largely resolved (Figure S4b).

We then aggregated reporter intensities to protein-level intensities, requiring at least two PSMs per protein. The majority of protein-level quantification estimates did not involve intensities below the notch, with a maximum of 63 proteins (1.7%) having 25% or greater ion intensities below the notch in any given tag (Figure S5a).

To formally test the impact of the notch on the detection of peptide or protein differential abundance, we used *limma* to detect differential abundance, with and without prior filtering to remove PSMs containing intensities at or below the notch. The accuracy for identification of changes in intensity was measured by the F1 score, the harmonic mean of precision, and recall. Fold-change estimates were very slightly closer to ground truth without notch filtering, but the difference was negligible (Figure S5b). For example, median fold changes for *S. cerevisiae* proteins in the 6× vs 1× comparison decreased from 4.963 to 4.949 (Figure S6a). Crucially, notch filtering did not improve the F1 score for the most difficult to identify changes (*H. sapiens* 2× vs 1×), which was 0.245 and 0.246, respectively, with and without notch filtering (Figure S6b). Similarly, for the easier to detect fold changes, F1 was not significantly affected by notch filtering.

To ensure that our observations were not dependent on the PSM filtering, we varied the thresholds for maximum interference (10, 50, 100%), minimum average signal/noise (0, 10), and minimum Sequest Delta CN score (0, 0.2, 0.5). Each combination of PSM filtering thresholds was repeated with and without removing PSMs with intensities below the notch (“notch filtering”). Thus, in total, 36 PSM filtering regimes were compared, representing a comprehensive set of PSM filtering schemas. Regardless of the thresholds used, notch filtering had a negligible impact on fold-change estimates or the F1 score while always reducing the number of proteins that could be interrogated (Figure S6). Thus, removing PSMs with intensities below the notch does not appear to be beneficial and may even reduce sensitivity in differential protein abundance experiments.

Finally, we repeated the differential abundance testing at the peptide level. While differential abundance testing is more typically performed at the protein level, there are experiments where peptide-level testing is more appropriate, including in the analysis of post-translational modifications.^12^ Given fewer reporter ion intensities are used to quantify peptides, we expected a more significant impact from the notch filtering. However, no improvements in the fold changes were observed (Figure S5c) and the increase in the F1 score was typically 0.001–0.005 and thus too slight to justify notch filtering (Figure S7).

To inspect the impact of the notch filtering on the estimated fold changes more directly, we considered proteins where at least one PSM was removed in the filtering and compared the fold changes with and without notch filtering. As previously described, the proportion of proteins with a PSM intensity below the notch is relatively low. When using stringent PSM filtering, just 120 protein fold changes are affected by the notch filtering, of which 116 are yeast proteins. The clear majority of fold changes are further from the ground truth when notch filtering is used (Figure 2D). This observation is counter-intuitive given that intensities below the notch are predominantly underestimated, suggesting their removal should yield more accurate fold-change estimates. However, since TMT fold-change estimates are always compressed to some extent,^5,7^ the underestimation of very low intensities below the notch leads to overestimated fold changes and can therefore act to counter the ratio compression. While this is not a justification for retaining the notch to improve fold-change estimates, it further underlines the negligible impact the notch should have on typical proteomics experiments, where higher-level features are typically summarizations of multiple independent PSMs.

An important caveat to our observations is that our benchmark experiment possessed a typical notch prominence and was designed to mimic a simple experiment to detect differential protein abundance. It is possible that in datasets with a more prominent notch, the underestimated tag intensities could become more problematic. Additionally, the notch could conceivably be more problematic where accurate quantification of the ratios between tags is more important than identifying significant differences between treatment groups. If the presence of a prominent notch was found to be detrimental in specific applications, we expect that imputing values within and below the notch will rectify this, though careful consideration of the imputation method and parameters is required.^13^ Setting aside these caveats, we believe this artifact in existing Orbitrap Fusion, Lumos, and Eclipse quantification data can be safely ignored for routine proteomics experiments, but we recommend Orbitrap users update to Tune 3.0 or subsequent versions.

## Experimental Section

### Cell Culture and Harvest

*S. cerevisiae* (EOROSCARF; BY4742 (MATα his3Δ1 leu2Δ0 lys2Δ0 ura3Δ0)) were inoculated into YPD media (Bacto-peptone, yeast extract, 50% glucose) at 32 °C with constant agitation. Cells were collected by centrifugation when grown to an optical density OD_600_ ∼0.5, corresponding to the exponential phase, and snap-frozen in liquid nitrogen until lysis. Cell pellets were resuspended in lysis buffer (200 mM HEPES (pH 8.5), 8M urea, 0.2% SDS) supplemented with protease and phosphatase inhibitors (Roche cOmplete mini EDTA-free protease inhibitor cocktail; 11873580001, Roche PhosSTOP; 4906845001) and mechanically disrupted with glass beads (MERCK; G8772) using a FastPrep-24 5G (MP Biomedicals SKU; 116005500) with the manufacturer’s pre-defined program for yeast sample lysis (40 s mixing at 40 m/s). The lysate was centrifuged to pellet cell debris and the supernatant was transferred to a fresh tube.

Human epithelial bone osteosarcoma, U-2 OS (ATCC HTB-96) cells were cultured and incubated in McCoy’s 5A (Gibco; 16600082) supplemented with 10% FBS at 37 °C in humidified conditions with 5% CO_2_ and tested to confirm the absence of Mycoplasma. Cells were harvested at ∼90% confluence by scraping directly from the plate in chilled lysis buffer (as used in yeast lysis) and sonicated on high setting for a total of 15 min (30 s cycles) at 4 °C using a Bioruptor Plus. The lysate was centrifuged to pellet cell debris and the supernatant was transferred to a fresh tube.

### Reduction, Alkylation, and Digestion

Nuclease enzyme (Millipore Benzonase Nuclease HC; 71206-3) was added to cell lysates to breakdown interfering DNA before quantification of protein concentration by the BCA assay (Thermo Scientific 23225) according to the manufacturer’s instruction. Disulfide bonds in the lysates were reduced with 15 mM dithiothreitol (DTT) for 1 h at 37 °C, followed by alkylation with 55 mM iodoacetamide (IAA) for 1 h at room temperature in the dark. To remove urea and other substances that could interfere with digestion, lysates were precipitated using chilled 50:50 ethanol:acetone overnight at −20 °C. The resulting protein pellets were then resuspended in 100 mM HEPES (pH 8.5) and sonicated for a total of 15 min (30 s cycles) to break up the pellets. Proteins were digested in a two-step digestion process with the 100:1 protein/protease ratio of Lys-C (Promega; V1671) at 37 °C for 4 h, followed by 100:1 trypsin digestion (Promega; V5111) at 37 °C overnight.

### Peptide Mixing and TMT Labeling

Peptide concentrations were measured using a fluorometric peptide assay (Pierce; 23290) before preparing human/yeast species mixes to specified ratios. For each tag, a sample was prepared containing a specified quantity of yeast and human peptides. Five micrograms of yeast peptides was used for tags 126, 127N, 127C, and 128N, 10 μg for 128C, 129N, and 129C, and 30 μg for 130N, 130C, and 131. The samples were made up to 100 μg using human peptides, e.g., 95 μg for 126, 127N, 127C, and 128N. Samples were then TMT-labeled (Thermo Scientific; 90406), according to the manufacturer’s instructions, pooled, and lyophilized.

### Peptide Clean Up and Offline Pre-fractionation

The multiplexed sample was desalted using solid phase extraction (SPE) with a C18 cartridge (Waters SepPak; WAT054955) by binding and washing peptides with 0.1% trifluoroacetic acid (TFA) and eluting desalted peptides with 70% acetonitrile/0.05% acetic acid. Peptides were separated using a basic pH reverse-phase liquid chromatography (RP-LC) on an Acquity UPLC system with a diode array detector (210–400 nm) to monitor elution profiles. Peptides were eluted with an Acquity UPLC BEH C18 column (2.1 mm ID × 150-mm; 1.7 μm particle size) (Waters; 186002353) over a 50 min linear gradient from 5 to 75% acetonitrile in ammonium formate (pH 10.0) at a flow rate of 0.244 mL/min. A total of 34 fractions were taken from the elution gradient and concatenated into 15 fractions. Samples were subsequently dried and solubilized in 0.1% formic acid.

### Mass Spectrometry Data Acquisition

TMT-labeled samples were analyzed using a Dionex Ultimate 3000 RSLC nanoUPLC (ThermoFisher Scientific Inc., Waltham, MA) system online with an Orbitrap Lumos mass spectrometer (ThermoFisher Scientific Inc., Waltham, MA) and data were collected using both Tune 2.1 and 3.4 version. Peptides were loaded onto a trap-column (Thermo Scientific PepMap 100 C18, 5 μm particle size, 100A pore size, 300 μm i.d. × 5 mm length) and separation of peptides was performed by C18 reverse-phase chromatography at a flow rate of 300 nL/min and a Thermo Scientific reverse-phase nano Easy-spray column (Thermo Scientific PepMap C18, 2 μm particle size, 100A pore size, 75 μm i.d. × 50 cm length). All samples were acquired in a 120 min run applying data acquisition using synchronous precursor selection MS3 (SPS-MS3).^8^ Analytical chromatography consisted of Buffer A (HPLC H_2_O, 0.1% formic acid) and Buffer B (80% ACN, 0.1% formic acid). 0–3 min at 2% buffer B, 3–93 min linear gradient 2–40% buffer B, 93–100 min linear gradient 40–90% buffer B, 100–104 min at 90% buffer B, 104–105 min linear gradient 90–2% buffer B, and 105–120 min at 5% buffer B.

All *m*/*z* values of eluting peptide ions were measured in an Orbitrap mass analyzer, set at a resolution of 120 000, and were scanned between *m*/*z* 380 and 1500. Data-dependent MS/MS scans (3 s duty cycle time) were employed to automatically isolate and fragment precursor ions using collisional-induced dissociation (CID) (normalized collision energy of 35%). Only precursors with charges between 2 and 7 were selected for fragmentation, with an AGC target of 10 000 and a maximum accumulation time of 50 ms. Precursor isolation was performed by the quadrupole with the 0.7 *m*/*z* transmission window. MS2 fragments were measured with the Ion Trap analyzer. The dynamic exclusion window was set to 70 s. SPS ions were all selected within the 400–1200 *m*/*z* range. AGC targets and maximum accumulation times were set to 50 000 and 120 ms, respectively. Ten co-selected precursors for SPS-MS3 underwent Higher energy Collisional-induced Dissociation (HCD) fragmentation with 65% normalized collision energy and were analyzed in the Orbitrap with a nominal resolution of 50 000. Data was acquired with equivalent parameters for both versions of Tune, 2.1 and 3.4.

### Mass Spectrometry Data Analysis

Raw data were viewed in Xcalibur v3.0.63 (ThermoFisher Scientific), and data processing was performed in Proteome Discovered v2.4 (ThermoFisher Scientific). Reference *H. sapiens* and *S. cerevisiae* FASTA databases containing all review UniProt/Swiss-Prot entries were downloaded from www.uniprot.org on April 2018 and June 2020, respectively. The raw files were submitted to a database search using PD with Sequest HF algorithm using the concatenated reference databases and the Common contaminant database.^14^ The peptide and fragment mass tolerances were set to 10 ppm and 0.5 Da, respectively. Static modification carbamidomethyl on cysteine was applied as well as TMT-6plex tagging of lysines and the peptide N terminus. Oxidation of methionine and deamidation of asparagine and glutamine were included as variable modifications and up to two missed cleavages were allowed. The percolator node was used for false discovery rate estimation and only rank one peptide identifications of high confidence (FDR <1%) were accepted.

Previously published data^4,11^ was downloaded from pride accessions PXD011254 and PXD007683 in raw format. Only TMT data was reanalyzed from PXD007683. Raw data was reanalyzed as indicated above, except that the database searching was performed with Mascot server (ver. 2.4, Matrix Science; using the same parameterization, as described above). The reference proteomes for *H. sapiens* (UP000005640) and *S. cerevisiae* (UP000002311) were downloaded in FASTA format on 17 January 2020.

### Data Processing and Analysis

PSM-level quantification was exported from Proteome Discoverer in text format. Downstream processing, filtering, analysis, aggregation, and visualization were performed using R^15^ v4.0.3 “Bunny-Wunnies Freak Out” and the tidyverse,^16^ MSnbase,^17^ and camprotR (https://github.com/CambridgeCentreForProteomics/camprotR) packages.

PSM quantifications were filtered to remove potential contaminating proteins using the cRAP database.^14^ PSMs without a master protein(s) were excluded. PSMs that could have originated from both yeast and human proteins (regardless of their master protein assignment) were excluded. Based on a visual assessment of the tag intensity distributions, the approximate boundaries of the notch were denoted as 4.25 and 5.75.

For the prediction of expected reporter ion intensities with the reanalyzed data, PSMs with the same sequence within an experiment were grouped, excluding groups with only one PSM, or where the highest-intensity PSM contained missing values. Intragroup intensity adjustment factors were then calculated, representing the intensities of each PSM relative to the most intense PSM, using only the channels without missing values. The highest-intensity PSM was then normalized by dividing each tag intensity by the total PSM intensity, such that the total normalized intensity was 1. These normalized intensities were then multiplied by the adjustment factors to yield tag intensity predictions for each PSM in the group, except the highest-intensity PSM.

To explore the relationship between tag intensities in the benchmark dataset, individual tag intensities were compared to the mean value for a comparator group of tags. A generalized additive smoothing model for the relationship between tag intensity and intensity difference was fitted using the default model for the ggplot function geom_smooth with method=“gam”, namely, the gam function in mgcv, with options “formula = y ∼ s(x, bs = “cs”)” and “method = “REML”.”

Prior to PSM filtering, to remedy any small difference in the total tag intensity in each channel, PSM intensities were log center-median normalized, before exponentiating back to the untransformed values.

PSM filtering was performed using 4 PSM metrics: (1) Sequest Delta CN score, which represents the normalized difference in the spectrum matching scores between the top-ranked and second-ranked peptides, calculated as Delta CN = (rank 1 score - rank 2 score)/rank 1 score, and is thus bounded between 0 (no difference) and 1 (no rank 2 score). Because our TMT-labeled samples contain a mixture of human and yeast proteins, there is an increased risk of incorrect spectrum matches. As the proteins from the two species have different fold changes between tags, any incorrect spectrum match has the potential to lead to very incorrect fold-change estimates. Hence, the introduction of an additional layer of filtering by Delta CN score. (2) Co-isolation, as reported by Proteome Discoverer, calculated as 100 × (1 – (precursor intensity in isolation window/total intensity in isolation window)). (3) Average reporter signal/noise, representing the average tag intensity for the PSM. (4) Presence of values within or below the notch, where the upper boundary of the notch was used to identify PSMs with any values below this threshold. The following values were used to filtering PSMs according to these metrics: (1) Sequest Delta CN score >0, 0.2, or 0.5, (2) Co-isolation <100, 50, or 10%, (3) Average reporter S/N >0 or 10, and (4) Notch PSMs retained or removed. All combinations of filtering thresholds were performed, yielding 36 sets of filtered PSMs.

Aggregation to protein-level intensities involved removal of PSMs with missing values, removal of proteins without at least two PSMs, and summation of PSM-level tag intensities. Aggregation of PSM intensities to peptide-level intensities proceeded in the same manner but demanded at least two PSMs per peptide sequence.

### Statistical Analysis

To identify peptides and proteins with significant differential abundance, we used *limma v3.44.3*.^18^*Limma* was run with default settings, with the exception that we set trend=TRUE for the eBayes function call so that the prior variance was dependent on the trend between feature intensity and observed variance. Protein intensity was modeled to depend on condition, with results extracted for the contrast between 1× vs 2× and 1× vs 6× tags. *P* values were adjusted for multiple testing within each PSM filtering schema, using the Benjamini–Hochberg False Discovery Rate (FDR) procedure.^19^ Features with FDR <1% were deemed to have significantly different intensity.

Yeast proteins with a significant increase in intensity and human proteins with a significant decrease in intensity were deemed true positives (TP). Vis versa, significant changes in the opposite direction were deemed false positives (FP). Proteins without significant changes in intensity were deemed false negatives (FN).

F1 scores were calculated as the harmonic mean of recall (TP/(TP + FN)) and precision (TP/(TP + FP)). The F1 score is bounded [0–1], with a higher score indicating a better balance between precision and recall. No proteins were deemed true negatives (TN) since all proteins should have a change in intensity.

### Extracting Tune Version from PRIDE Submissions

Dataset identifiers for 8274 studies using Orbitrap were obtained from ProteomeCentral (http://proteomecentral.proteomexchange.org/cgi/GetDataset) by searching for entries with “Orbitrap” in the instrument text and dated no later than 31 December 2021. These were further narrowed down to 2024 studies in the PRIDE repository with “Orbitrap Fusion,” “Orbitrap Fusion Lumos”, or “Orbitrap Eclipse” listed as an instrument since these share the same series of signal acquisition software. Studies with multiple instruments listed were ignored to avoid incorrectly asserting the version of Tune used. For each study, the smallest.raw file was downloaded and the meta information was extracted using the R package rawDiag,^20^ including the version of the acquisition software, Tune. For 269/2024 studies, the Tune version was not extracted either because the instrument detailed in the.raw file was not one of the above Orbitraps, no.raw files were found, the downloaded.raw file could not be parsed by rawDiag, or the.raw file URLs did not permit downloading.

## Supporting Information Available

Figure S1: Tune version parsing and reporter ion intensity distributions. Figure S2: characterization of the reporter ion intensities around the notch; Figure S3. reporter ion intensities in benchmark datasets. Figure S4: reporter ion intensity and fold changes with Tune 3.4. Figure S5: impact of notch filtering on fold changes. Figure S6: summary of limma analysis of protein-level intensities. Figure S7: summary of limma analysis of peptide-level intensities (PDF)
The mass spectrometry proteomics data have been deposited to the PRIDE Archive (http://www.ebi.ac.uk/pride/archive/) via the PRIDE partner repository with the dataset identifier PXD027248.All analyses and results are publicly available from the github repository https://github.com/CambridgeCentreForProteomics/notch, alongside the peptide spectrum match (PSM)-level quantification from Proteome Discoverer, and reference FASTA databases used. Release v0.3 of the repository used for this manuscript is archived with DOI: 10.5281/zenodo.5885619.

## Notes

This research was funded in whole, or in part, by the Wellcome Trust grant 110071/Z/15/Z. For the purpose of open access, the author has applied a CC BY public copyright license to any Author Accepted Manuscript version arising from this submission. This work is licensed under a Creative Commons Attribution 4.0 International License.
