# Supplementary material for: Prior Signal Acquisition Software Versions for Orbitrap Underestimate Low Isobaric Mass Tag Intensities, Without Detriment to Differential Abundance Experiments
Source: ACS Meas Sci Au. 2022 Mar 9;2(3):233–40. doi: 10.1021/acsmeasuresciau.1c00053 (PMC9204819; doi:10.1021/acsmeasuresciau.1c00053)
Supplement: Supplementary file 1 — tg1c00053_si_001.pdf [file tg1c00053_si_001.pdf]

Supporting information for: Prior Signal Acquisition Software Versions for Orbitrap Underestimate Low Isobaric Mass Tag Intensities, Without Detriment to Differential Abundance Experiments

Tom S Smith[1*], Anna Andrejeva[2], Josie Christopher[2], Oliver M. Crook[3], Mohamed Elzek[3], Kathryn S Lilley[2]

1. University of Cambridge, MRC Toxicology Unit, CB2 1QR

2. University of Cambridge, Department of Biochemistry, CB2 1QW

3. University of Oxford, Department of Statistics, OX1 3LB

* Corresponding author. Email: tss38@cam.ac.uk

Table of contents

Figure S1 .... S-1

Figure S2 .... S-2

Figure S3 .... S-3

Figure S4 .... S-4

Figure S5 .... S-5

Figure S6 .... S-6

Figure S7 .... S-7

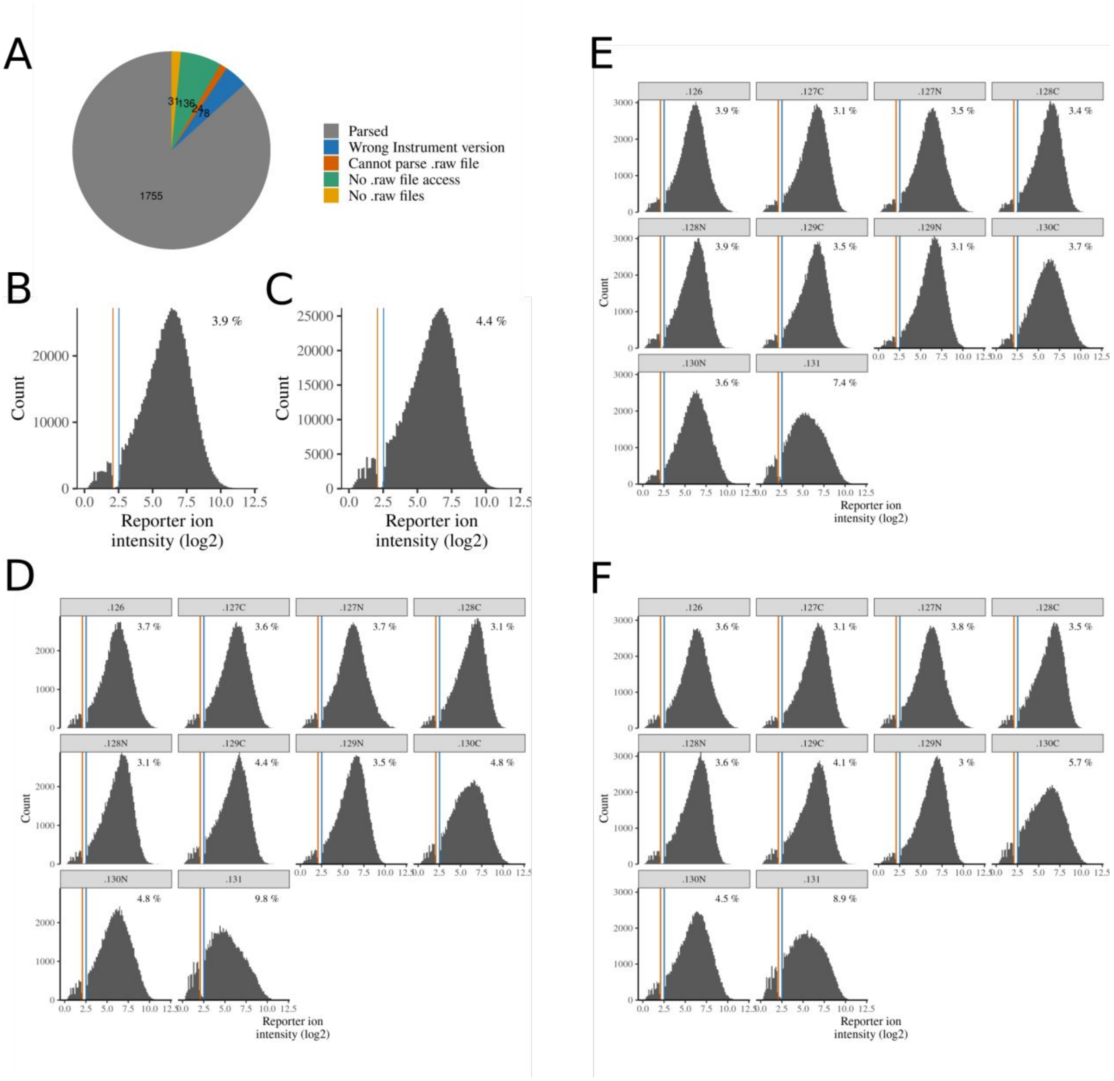

Figure S1.(A) The Tune version could be 'Parsed' from 1283 studies, with the remaining not accessible for the reasons described. (B-C) The distribution of ion signals for TMT reporters for U-2 OS LOPIT-DC replicate 2 (B) and replicate 3 (C). The approximate boundaries of the notch region are denoted by vertical lines. The percentage of tag intensities below the upper boundary of the notch is stated in the top right corner. (D-F) The distribution of ion signals for each individual TMT reporter; replicate 1 (D), replicate 2 (E) and replicate 3 (F).

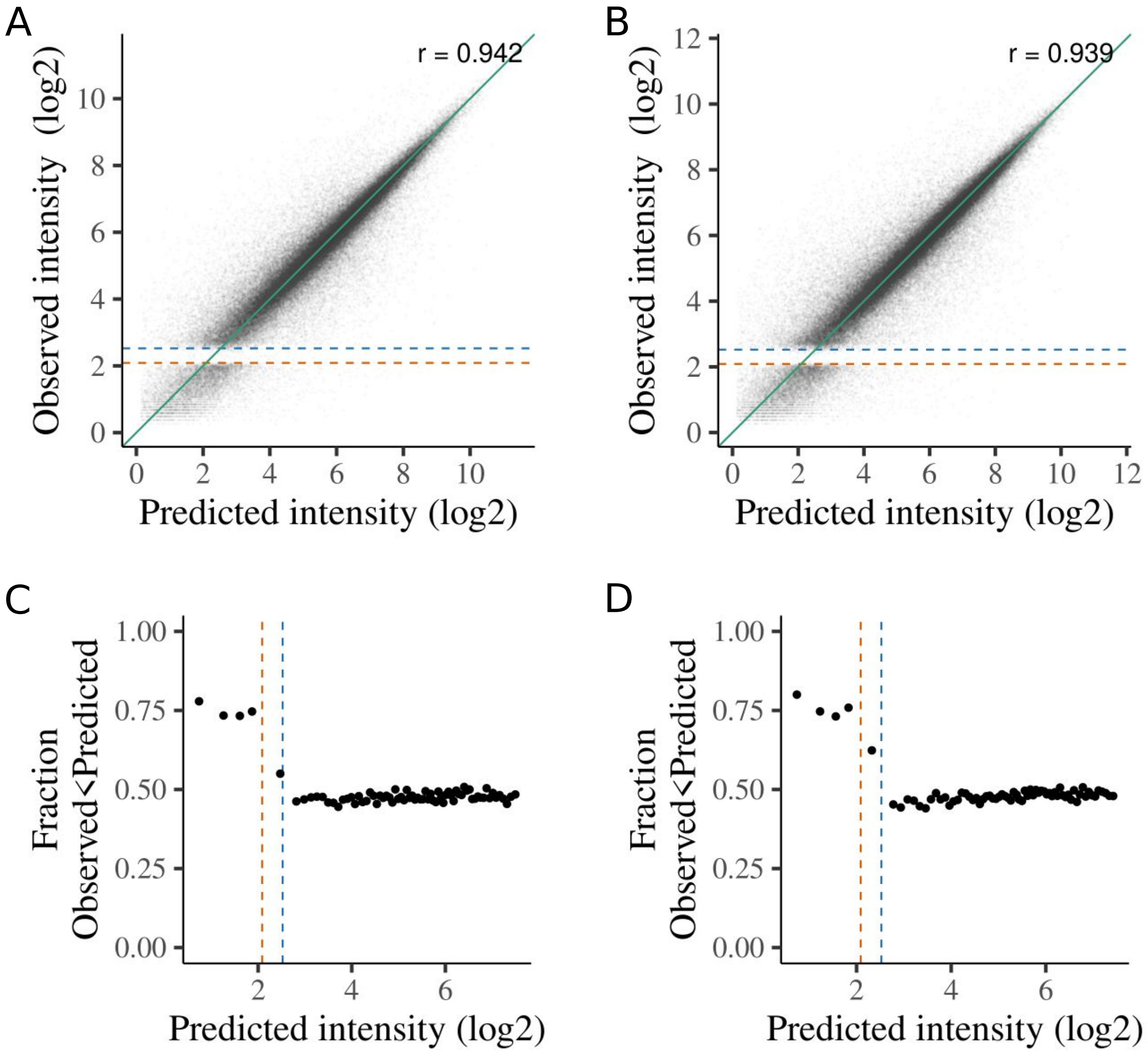

Figure S2: Characterisation of the reporter ion intensities around the notch in U-2 OS LOPIT-DC (A-B) Observed tag intensities vs predicted tag intensities for replicate 2 (A) and replicate 3 (B). Green line is equality. Pearson product-moment correlation coefficient shown in the top right corner. (C-D) Fraction of observed reporter ion intensities that are below the prediction for replicate 2 (C) and replicate 3 (D). Observed tag intensities below the notch are predominantly underestimates relative to the prediction.

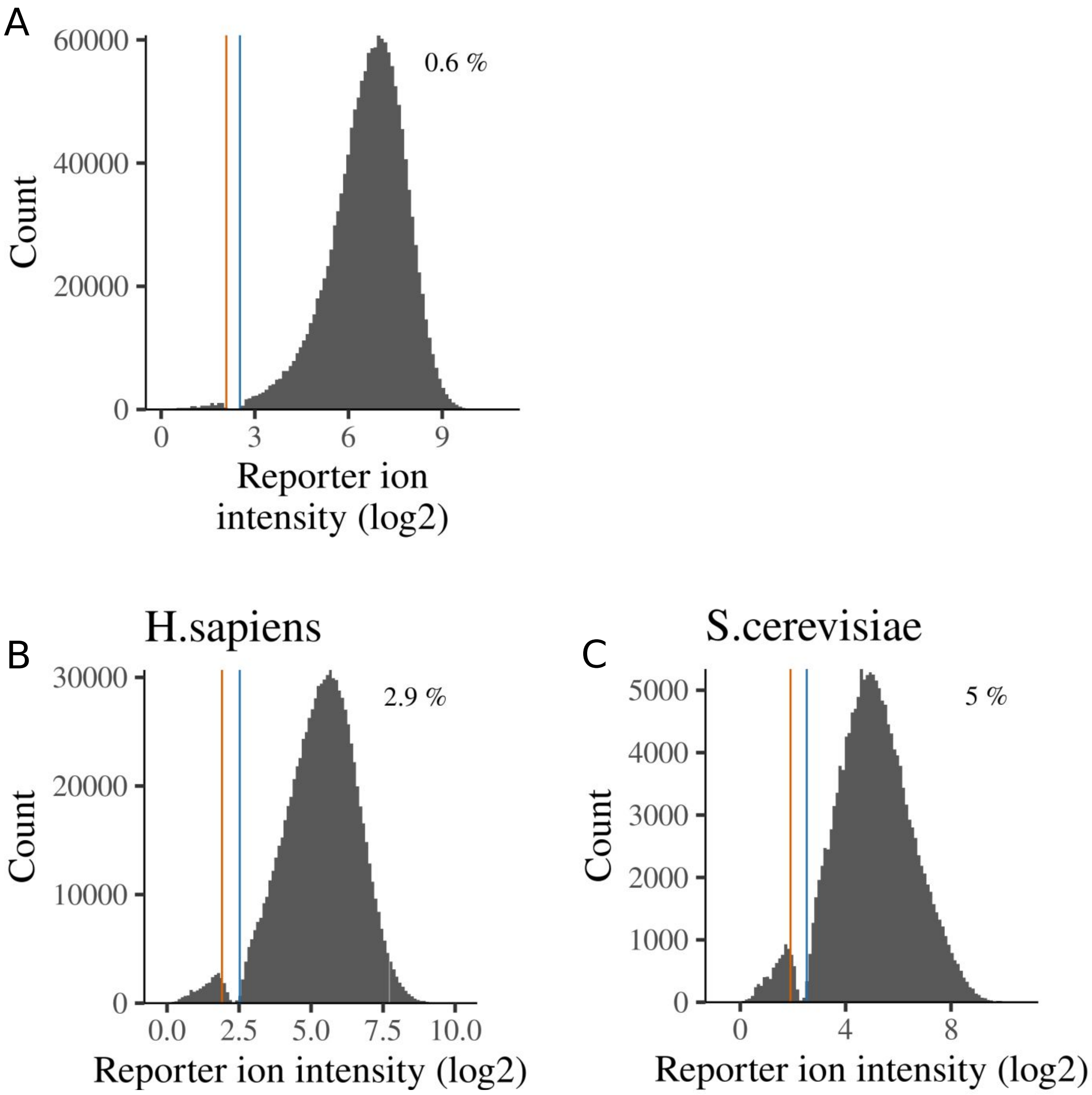

Figure S3. Distributions for reporter tag intensities in O'connell et al data (A; [4]) and the benchmark dataset, considering only human (B) or yeast (C) proteins. The percentage of tag intensities below the upper boundary of the notch is stated in the top right corner.

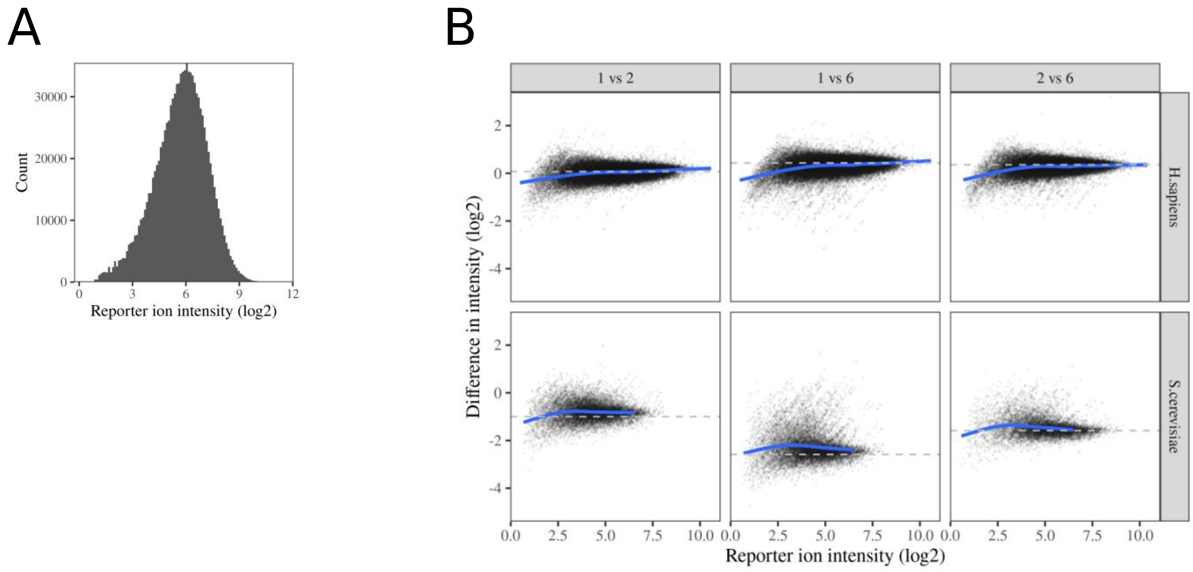

Figure S4 Reporter ion intensity and fold-changes for benchmark dataset when using Tune 3.4.(A) The distribution of reporter ion intensities for TMT reporters. No notch is observed. (B) The difference between a single tag intensity and the mean tag intensity for a comparator group of tags. The ground truth is denoted by a dashed horizontal line. The blue line presents a generalized additive smoothing model for the relationship between tag intensity and intensity difference.

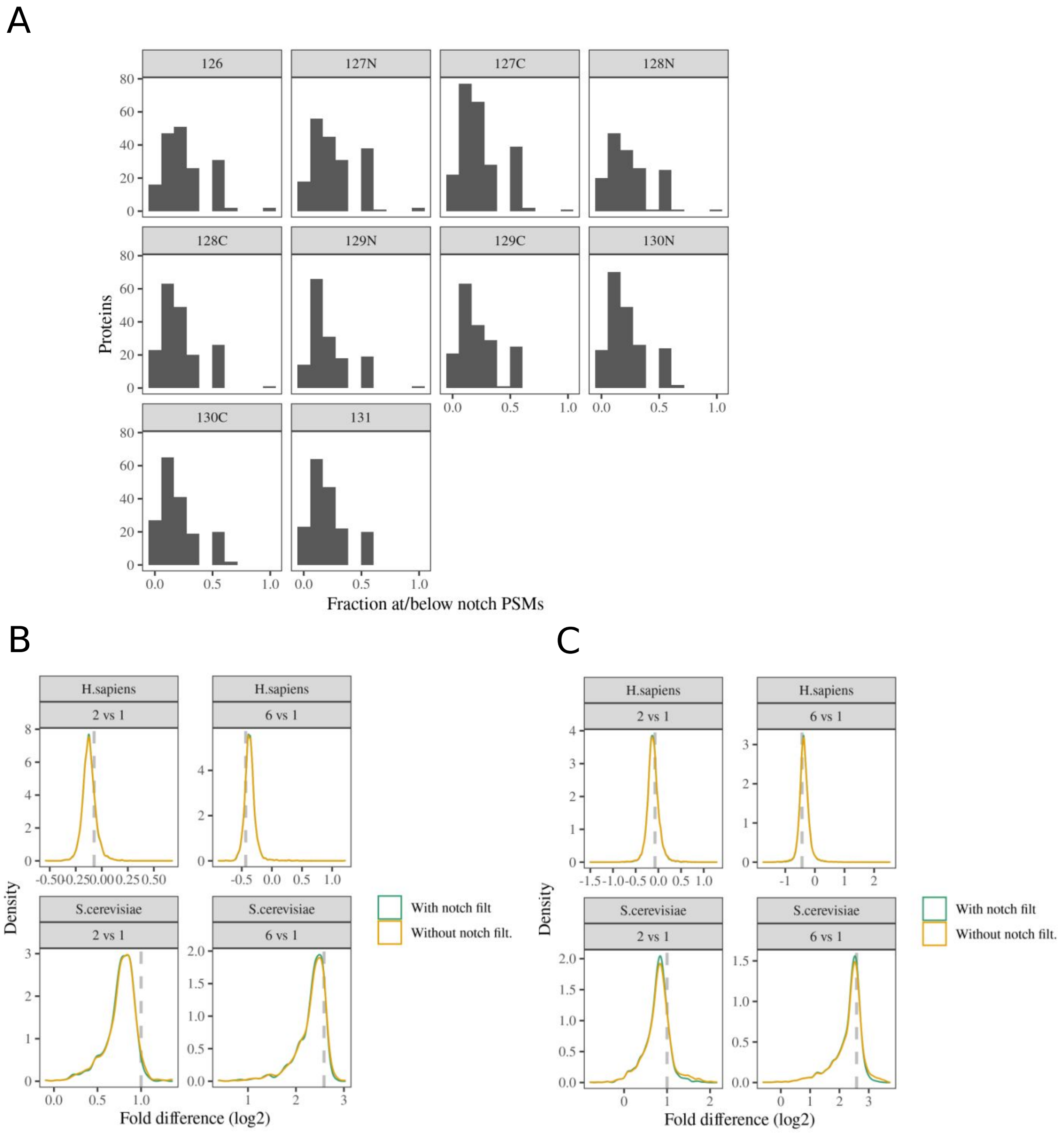

Figure S5. (A) Tallies for the fraction of the aggregated reporter ion intensities at or below the notch for each protein. Proteins with no intensities at or below notch are not tallied. (B-C) Observed fold-changes between tag groups, with and without notch filtering, for proteins (B) and peptides (C). Facet text indicates which spike-in groups are being compared (see Figure 2A for experimental design). Vertical dashed line indicates the fold-change ground truth.

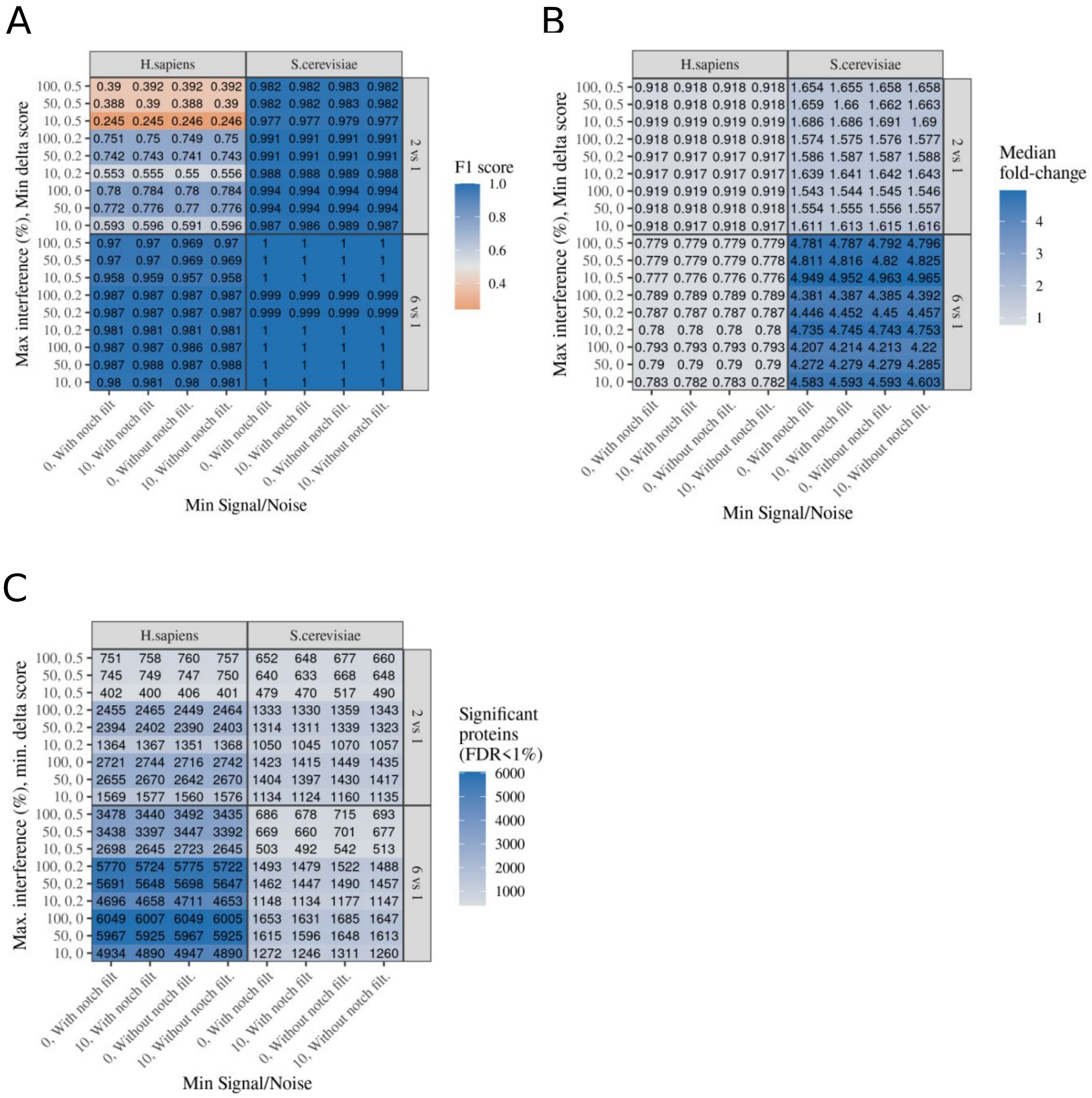

Figure S6. Summary of limma analysis of protein-level intensities. Limma analysis was performed on datasets generated from multiple different PSM filtering schemas, as indicated on the x-axis and y-axis. Each axes describe the combination of two filtering parameters. 'Min Signa/Noise' is the minimum average signal/noise for the PSM. The x-axis labels further describes whether PSMs containing any intensities at or below the notch were removed. 'Max interference (%)' describes the maximum allowed interference/co-isolation. 'Min delta score' describes the minimum required delta score. (A) Accuracy of differential protein intensity detection, presented as the F1 score (harmonic mean of precision and recall). Only proteins present with all PSM filtering schemas were included (B) Median fold change between groups of tags. Only proteins present with all PSM filtering schemas were included. (C) The number of proteins with significantly different intensity.

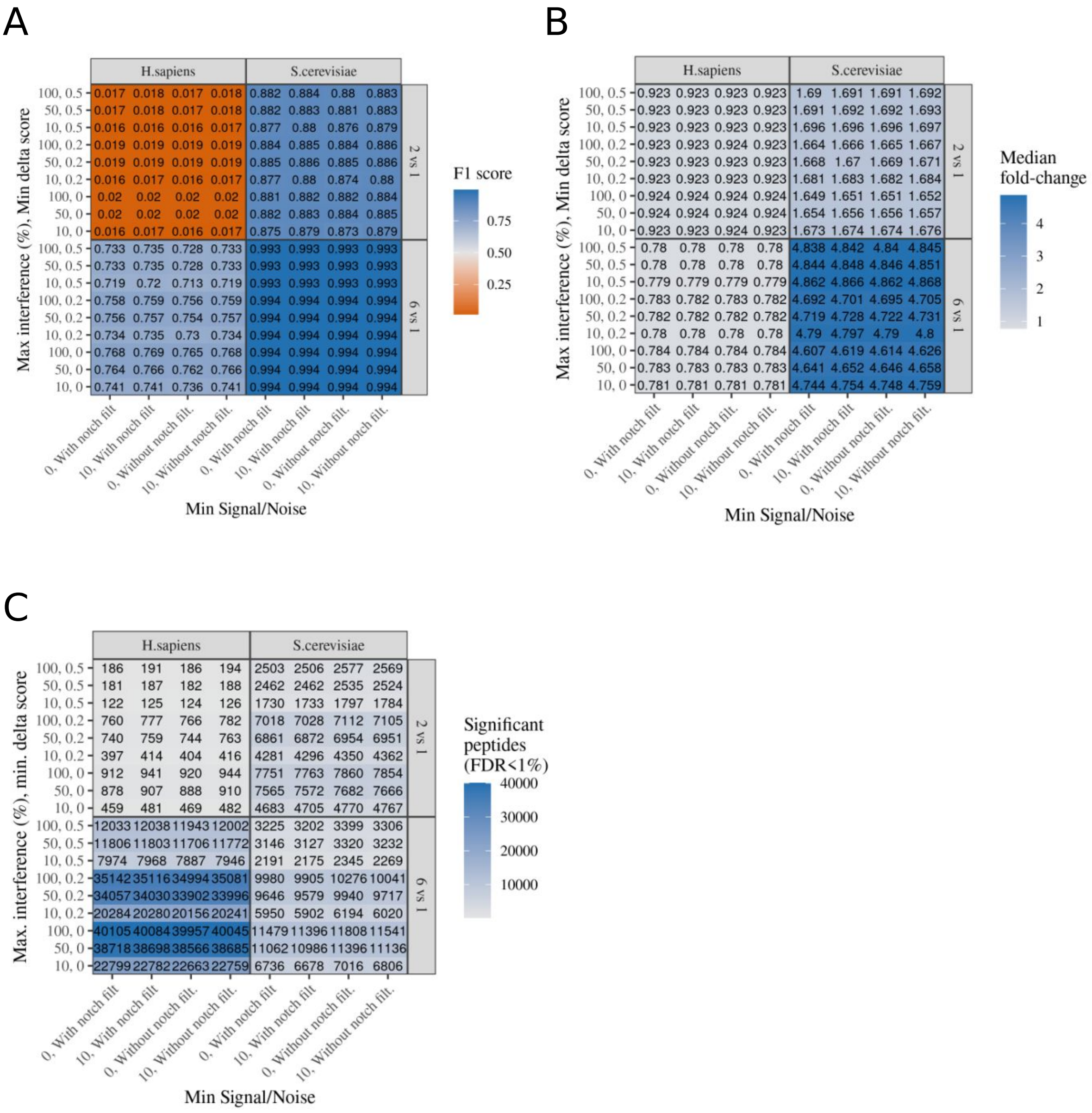

Figure S7. Summary of limma analysis of peptide-level intensities. Limma analysis was performed on datasets generated from multiple different PSM filtering schemas, as indicated on the x-axis and y-axis. Each axes describe the combination of two filtering parameters. 'Min Signa/Noise' is the minimum average signal/noise for the PSM. '+ Notch' describes whether PSMs containing any intensities at or below the notch were removed. 'Max interference (%)' describes the maximum allowed interference/co-isolation. 'Min delta score' describes the minimum required delta score. (A) Accuracy of differential protein intensity detection, presented as the F1 score (harmonic mean of precision and recall). Only peptide present with all PSM filtering schemas were included (B) Median fold change between groups of tags. Only peptide present with all PSM filtering schemas were included. (C) The number of peptides with significantly different intensity.
